# Functionalization of graphene: does the organic chemistry matter?

**DOI:** 10.3762/bjoc.14.177

**Published:** 2018-08-02

**Authors:** Artur Kasprzak, Agnieszka Zuchowska, Magdalena Poplawska

**Affiliations:** 1 Faculty of Chemistry, Warsaw University of Technology, Noakowskiego Str. 3, 00-664 Warsaw, Poland

**Keywords:** characterization, functionalization, graphene, modification, synthesis design

## Abstract

Reactions applying amidation- or esterification-type processes and diazonium salts chemistry constitute the most commonly applied synthetic approaches for the modification of graphene-family materials. This work presents a critical assessment of the amidation and esterification methodologies reported in the recent literature, as well as a discussion of the reactions that apply diazonium salts. Common misunderstandings from the reported covalent functionalization methods are discussed, and a direct link between the reaction mechanisms and the basic principles of organic chemistry is taken into special consideration.

## Introduction

**Figure 1:**
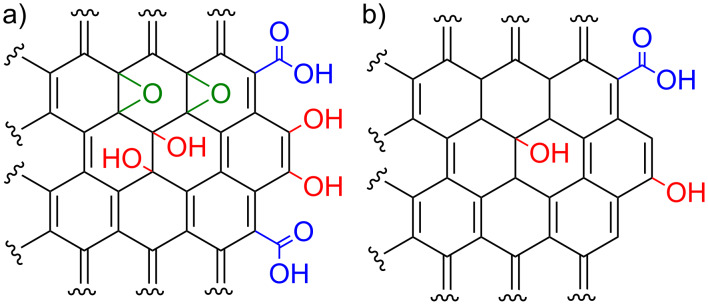
Partial structure [7–8] of the (a) graphene oxide (GO) and (b) reduced graphene oxide (RGO).

In 2004, Geim and Novoselov reported the first experimental isolation of the graphene sheet and the measurement of its properties [1]. Since then, the researchers have presented many different applications of this special carbon nanostructure [2–3]. Graphene oxide (GO) and reduced graphene oxide (RGO) have most commonly been investigated in terms of creating novel functional materials. GO is a product of oxidative exfoliation from bulk graphite [4]. GO’s structure (Figure 1a) comprises a large number of oxygen functionalities, including carboxyl (COOH), hydroxy (OH) and epoxide (see the moieties in green in Figure 1a) groups. Treating GO with high temperature or with reducing agents yields RGO (Figure 1b) [5]. As a result of the reduction process, the oxygen content in RGO is lower than that in GO, however, some oxygen groups are present in RGO’s structure [6].

To trigger some desired effects and to open new avenues for the application of GO and RGO, a chemical functionalization was conducted [9–12]. There are two approaches for the structural modification of GO and RGO: (i) the reaction of oxygen-bearing groups or (ii) the functionalization of sp^2^ carbons from the graphene sheet. The carboxyl groups contained in GO and RGO constitute important targets for structural expansion, as COOH moieties can be transformed into, e.g., amide- or ester-type linkages. On the other hand, the functionalization of sp^2^ carbons is most commonly performed utilizing a diazotization approach; in other words, the diazonium salt is generated from the corresponding aromatic amine (preparation of the reagent), and then the aryl radical is added to the graphene sheet.

The crucial parts of a significant number of the articles on the application of GO and RGO focus on the chemical functionalization of the graphene-family material. In fact, the structural modification of GO and RGO constitutes a key starting point of such research. Even though many studies on chemical functionalization have presented very interesting and novel applications of GO and RGO derivatives they also present yet common misunderstandings and inaccuracies. This work deals with common issues in the field of GO and RGO functionalization; it discusses the carboxyl-based approach and includes remarks regarding reactions that utilize diazonium salts. Direct links are provided to basic principles of organic synthesis, reaction mechanisms, and state of the art of organic chemistry. Point-by-point recommendations are also given for the proper application of organic chemistry principles in covalent functionalization of graphene-family materials. The chemistry of the reactive groups of graphene-family materials covers many areas of research, including colloid chemistry and interface science; nevertheless, the basic rules of organic chemistry should be regarded as playing a leading role in covalent functionalization.

## Review

### Reactions of carboxyl groups: amidation, esterification

**Figure 2:**
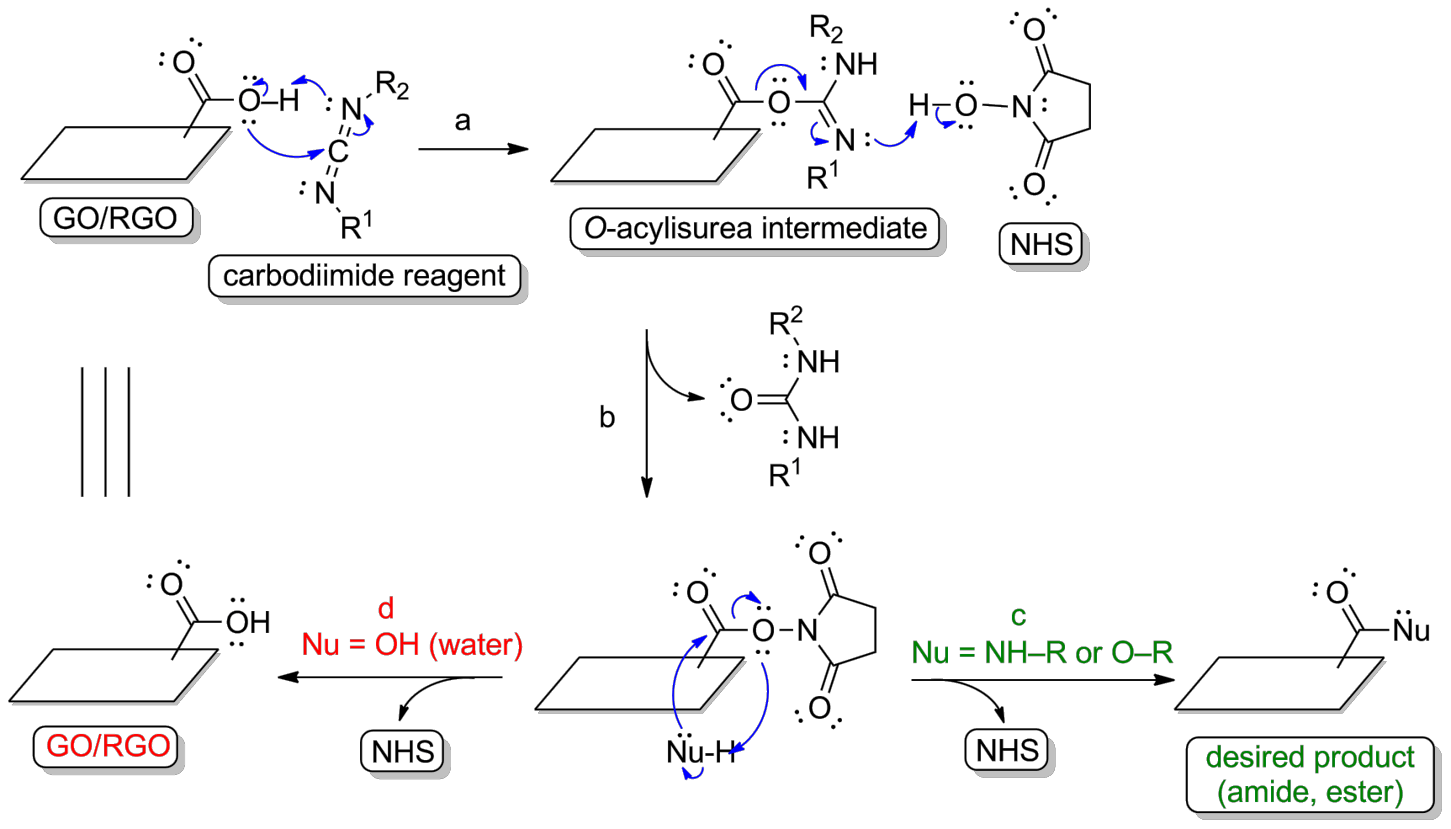
Mechanism of the amidation/esterification-type reactions with the GO/RGO using carbodiimide and *N*-hydroxysuccinimide activation: (a) activation of the carboxyl group with a carbodiimide reagent, (b) reaction with *N*-hydroxysuccinimide, (c) amidation/esterification-type reaction with the desired nucleophile, (d) reaction of the activated carboxyl group with water molecules.

A primary amine or a primary alcohol constitutes a nucleophilic partner in a reaction with a carboxyl group (COOH). As a result of derivatization, an amide or ester bond is formed between a graphene-family material and a given chemical. The nature of the derivatization of carboxyl functionalities onto the graphene sheet is directly associated with improvements in the reactivity of carboxyl moieties. Carboxyl groups are not as reactive as the corresponding acyl chlorides or anhydrides. Activating a carboxylic group is therefore a crucial step in improving its reactivity toward nucleophilic reagents. A common approach is to employ carbodiimide-promoted reactions [13–14]. As presented in Figure 2, step a, the first step of a carbodiimide-type conjugation involves the generation of an *O*-acylisourea intermediate, which is highly electrophilic and which bears the urea-based good leaving group. Such activation of carboxyl groups onto a graphene material makes these functionalities very reactive toward the nucleophilic reagents.

Strong nucleophiles such as primary aliphatic amines, are readily reactive toward activated carboxyl groups. However, the *O*-acylisourea intermediate is also reactive toward water molecules and in some cases may react slowly with the desired nucleophile. To increase the stability of the active *O*-acylisourea intermediate and to promote the creation of amide- or ester-type linkages, an additional coupling reagent is included in the process (Figure 2, step b). Doing this is especially important when the concentration of the nucleophilic reagent is very low. In the amine coupling, *N*-hydroxysuccinimide is a commonly applied additive. The resulting ester is more stable than the corresponding *O*-acylisourea intermediate. The stability increases the reaction rate with the target nucleophile and the formation of the desired amide bond (Figure 2, step c).

**Figure 3:**
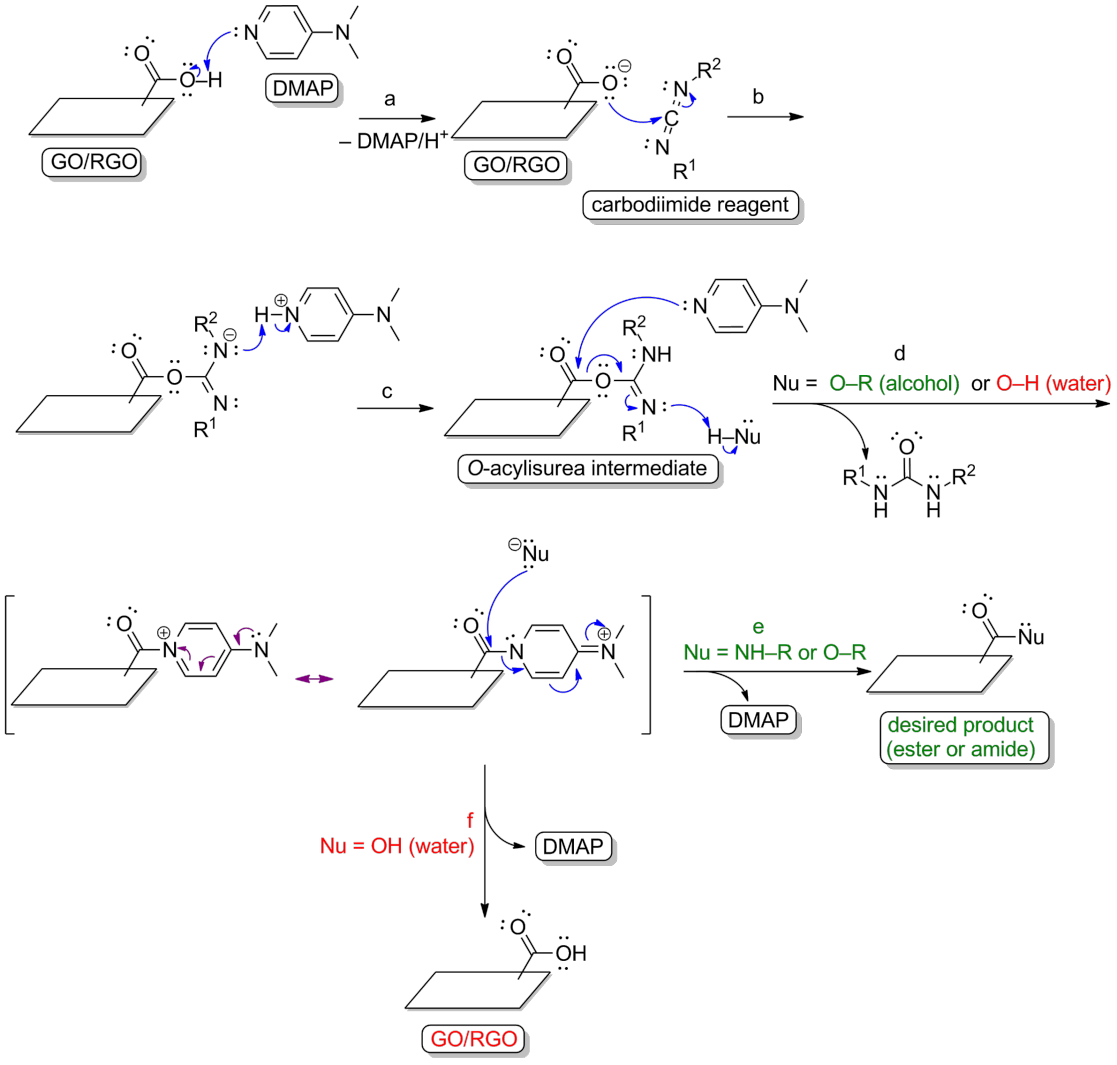
Mechanism of the Steglich esterification with the GO/RGO: (a) acid–base reaction of the carboxyl group with DMAP, (b) activation with a carbodiimide reagent, (c) reaction with DMAP, (d and e) desired reaction pathway (ester or amide bond formation), (d and f) reaction of the activated carboxyl group with water molecules.

On the other hand, reactions with alcohols proceed at slower rates, for the difference in the nucleophilicity of a primary alcohol and a water molecule is not as prominent as the difference found in amine coupling. Steglich esterification is a widely applied approach for ester bond formation [15]. 4-(*N*,*N*-Dimethylamino)pyridine (DMAP) is as an additive in the carbodiimide-coupling protocol. In the second step, DMAP forms active amide intermediates via a reaction with an *O*-acylisourea individual (Figure 3). This is because DMAP is a stronger nucleophile than the alcohol. This leads to the formation of the desired ester bond (Figure 3, step e). DMAP acts both as a nucleophile and an acyl transfer reagent and suppresses the side reactions.

**Figure 4:**
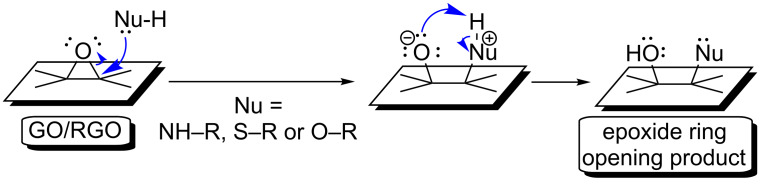
Mechanism of the epoxide ring opening reaction with the GO/RGO.

The structure of GO includes a number of epoxide moieties, which are also reactive toward the nucleophilic reagents. The epoxides’ opening by nucleophiles can therefore act as a competing side reaction in many coupling processes with the inclusion of graphene-family materials [9,16]. In other words, opening graphene-family material’s epoxides should be taken into account even when the crosslinker-based amidation or esterification approach is performed. The mechanism of opening graphene-family material’s epoxides is presented in Figure 4. This conversion involves a nucleophilic attack on the sp^3^ carbon, thus leading to the desired product. This functionalization route is simple, as it does not require coupling reagents. Strong nucleophiles (e.g., primary amines or thiols) react with epoxides more rapidly than do weak nucleophiles (e.g., like primary alcohols). This functionalization approach based on the epoxides’ opening enables the introduction of the reactive groups to the surfaces of graphene-family materials. The properties of these materials can thus be tuned, and the introduced functional groups can be utilized for further reactions [9,17–19].

**Figure 5:**
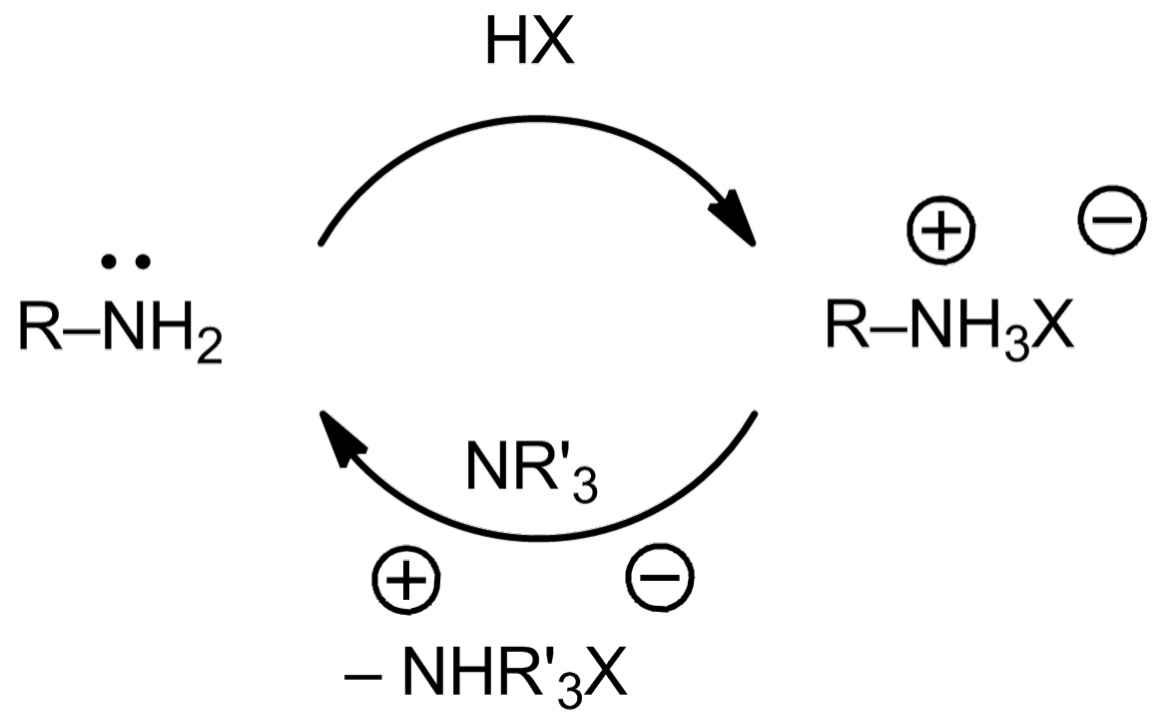
Generation of the free amine (nucleophile) from the corresponding amine hydrohalide using an acid–base reaction with the tertiary amine.

The literature features many examples of amide- or ester-bond formation with the inclusion of graphene-family materials [20–21]. However, some of the reported conjugation protocols do not uphold with the basic organic chemistry principles discussed above. Despite the interesting applications presented in these studies, important questions remain regarding the structure of the obtained materials. One common inaccuracy is a lack of the additives in the conjugation process, which further leads to a misleading material structure. For example, it is very confusing that in some cases the nucleophilic reagent in the coupling reaction is not a nucleophile at all. The most prominent example (beyond just in the graphene chemistry) is the reaction between carboxyl groups and the hydrohalides of the corresponding amines [22]. An amine hydrohalide is not nucleophilic because the lone pair of electrons on the nitrogen atom is involved in the formation of the hydrohalide individual. As visualized in Figure 5, when an amine hydrohalide is subjected to the discussed conjugation reaction, a tertiary amine (e.g., triethylamine) should be also included in the process. The tertiary amine’s role is to transform the amine hydrohalide into a free amine via the acid–base reaction. The free amine can then act as a strong nucleophile in the desired amidation process or can attack the epoxides of the graphene-family material. In a coupling with the inclusion of graphene-family material and amine hydrohalide some reactions or transformations may occur, but they are based on electrostatic adsorption or hydrogen bonding [23], rather than on covalent modification. If formation of the stable amide bond via a reaction between amine hydrochloride and the carboxyl group of GO is stated, then a reaction mechanism for such an unexpected process should be proposed. However, the infrared (IR) and NMR data [22] are not consistent in some cases: The research on IR spectra has suggested that some reactions exist between the amine component and GO’s epoxides, but the NMR indicated that both amide bond formations and nucleophilic attacks on the epoxides exist. This raises a question about the exact structure of the product.

The same conclusion about the structure of the obtained material applies for the derivatization of GO and RGO based on heating or mixing a carbon nanostructure with an amine or hydroxy-containing component [24–26]. For example [24], it is not clear whether the word “amination” refers to the formation of the amide (NH–CO) bond or to the reaction that the amine component has with the epoxy groups of GO, as divergent notes are included in this study’s results. The reaction of GO with diamines is a well-documented process (i.e., a nucleophilic attack on GO’s epoxides) [16,27], so the unexpected presence of an amide bond in the material, obtained via the reaction between *p*-phenylenediamine and graphene oxide with no additive (heating at 80 °C for 24 h), should be directly observed using IR spectroscopy. In this case, benzene rings were claimed to be introduced to GO via the presence of a strong absorption band located at ca. 1500 cm^−1^ (C=C stretching vibrations) in the product’s IR spectrum. Therefore, it is not clear why a similar, prominent adsorption band corresponding to the amide moiety was not observed in the spectrum, and this issue was not discussed. It is noteworthy that both a lower intensity of the absorption band coming from the C=O of GO (above 1700 cm^−1^) and a new absorption band in the wavenumber range of 1550 cm^−1^ to 1620 cm^−1^ can be directly inferred from the formation of either a hydrogen bond between primary amine and the carboxyl groups of GO or an inner salt formation (i.e., COO^−^NH_3_^+^). The presence of the amide bond in the functionalized GO was implied by the deconvolution of the C1s (287.8 eV for NH–C=O vs 289.1 eV for O–CO) and N1s (399.4 eV for N–C=O vs 400.3 eV for O–CO) peaks in the X-ray photoelectron spectroscopy (XPS), only. However, the C=O stretching vibrations in the amide groups can indeed be easily observed in the infrared spectrum of the functionalized graphene-family materials; thus, XPS should be regarded as a supporting analysis, as many researchers have demonstrated (see, e.g., [21,28–29]). Interestingly, for a reaction that includes water molecules and a hydroxy group-bearing compound (β-cyclodextrin) [25], the approach to the synthesis of the material has not been given in some cases; in other words, the researchers have not always discussed whether the process is based on the non-covalent adsorption of the reactant or whether it follows the nucleophilic addition to GO’s epoxides. On the basis of the presented analyses the RGO surface can be assumed to include the adsorbed β-cyclodextrin (RGO has been obtained via a reduction of GO using sodium borohydride). Finally, there is a misunderstanding regarding the structure of the product bearing the amide moiety, as the lack of evidence for the formation of such a linkage, should be highlighted [26]. The applied functionalization protocol for GO functionalization and, importantly, the product’s IR spectrum directly suggest a nucleophilic attack on the GO’s epoxides instead of amide bond formation (as stated in the figure). For the infrared spectrum of the functionalized GO, no strong absorption band was observed in the wavelength range of ca. 1680–1630 cm^−1^ (i.e., in the typical wavelength range for the C=O of amide bond). Moreover, the most prominent absorption band (located at 1587 cm^−1^) was surprisingly neither discussed nor assigned [26]. Only the absorption bands located at 1475 cm^−1^ (N–H stretching vibrations) and 1385 cm^−1^ (C–N stretching vibrations) were assigned. However, these features cannot be considered direct evidence for the formation of amide-type linkages. Importantly, no mechanism for generating the amide bond (if any such bond formed) using L-cysteine has been proposed or discussed. Most plausibly, the attack of cysteine’s highly nucleophilic sulfur on GO’s epoxides did occur in this case [30–31], based on the changes observed in the IR spectrum of the product. The material’s structure would include free amino and carboxyl groups forming the inner salt or hydrogen bonding, as the presence of absorption bands located in 1610–1587 cm^−1^ (asymmetric vibrations of C=O of –COO^−^ and N–H of NH_3_^+^) and 1475–1385 cm^−1^ (symmetric vibrations of C=O of –COO^−^ and N–H of NH_3_^+^) ranges indeed implies. The absorption band located at 3016 cm^−1^, interestingly, was assigned to the stretching vibrations of the alkyl chain (commonly observed at up to 2980 cm^−1^); this absorption band corresponds to the N–H stretching vibrations of NH_3_^+^. The further reaction with amphotericin B, which is a compound containing unsaturated bonds, was most plausibly a result of π–π stacking.

**Figure 6:**
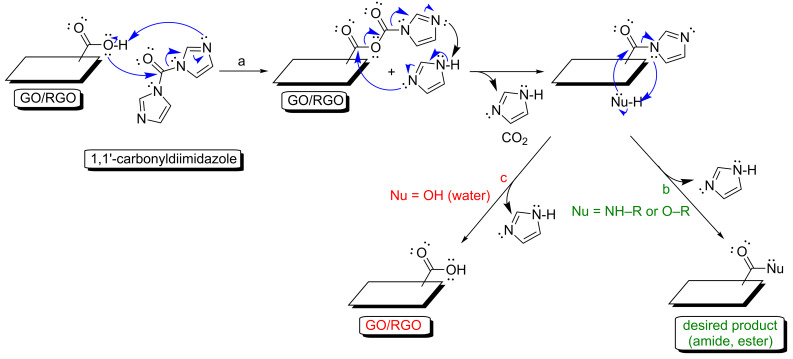
Mechanism of amidation/esterification-type reactions with the GO/RGO using 1,1’-carbonyldiimidazole: (a) activation of the carboxyl group with 1,1’-carbonyldiimidazole, (b) amidation/esterification-type reaction with the desired nucleophile, (c) reaction of the activated carboxyl group with water molecules.

As mentioned above in the discussion of reaction mechanisms, water molecules significantly lower the reaction rates for the desired nucleophiles. Importantly, water molecules also influence the hydrolysis of the activated carboxyl groups (Figure 2, step d and Figure 3, step f). Water should therefore be excluded to ensure the efficient esterification-type reactions with graphene-family materials. Aprotic organic solvents should instead be used as a reaction medium. It is obviously a barrier for the processes with the inclusion of GO or RGO, as these carbon nanostructures’ colloidal stability in aqueous solutions is higher than their colloidal stability in typical polar aprotic solvents such as dimethyl sulfoxide [32–33]. However, some researchers have reported successful attempts at functionalizing GO or RGO in organic solvent [34–37]. Surprisingly, some examples in which GO or RGO the esterification process is conducted with water as a reaction medium are also found in the literature [38–40]. For example [38], the formation of ester-type linkages have been found between the carboxyl group of GO and the hydroxy group of a sugar derivative via a water-based reaction mediated by the 1,1’-carbonyldiimidazole, which acts as the coupling reagent. The mechanism of this method is presented in Figure 6. The basic principles of this process are the same as for those of carbodiimide-mediated protocol (a reaction between activated ester and a nucleophile). As presented in Figure 6, steps b and c, there is significant concurrence in the nucleophilic attacks of the desired hydroxy group-bearing compound (Figure 6, step b) and water (Figure 6, step c) because of the similar nucleophilicity of these molecules. In other words, water only attacks the activated ester because of the much higher number of water molecules than primary alcohol molecules. The reaction rate of the desired esterification process is therefore extremely low. In practice, the formation of the ester bond is not favored under such conditions. In the study [38], it is highly possible that non-covalent and hydrogen bonding-dependent adsorption of glucose on the RGO surface results in a shift of the absorption bands coming from the C=O of RGO (from 1724 cm^−1^ to 1735 cm^−1^). Another consideration is that this process further influences the obtained material’s morphology, properties and thermal stability [41–42]. Additionally, some researchers have used the acid catalysis [39] or grinding-induced process [40] for the esterification reaction, and the changes in the spectra (e.g., IR, XPS) were not prominent enough to imply the formation of covalent ester-type linkages. In the grinding-induced case [40], it was not clear how the ester bond between RGO and hydroxypropyl-β-cyclodextrin would form, as the changes observed in the infrared spectra cannot be regarded as direct evidence for the formation of such linkages (IR analyses: C=O for carboxyl of GO: 1745 cm^−1^, C=O coming from ester bond stated for functionalized GO: 1639 cm^−1^). This unexpected statement was supported, not by further spectroscopic analyses, but by morphological studies (transmission electron microscopy (TEM) and atomic force microscopy (AFM)) only. Once again, the possible electrostatic adsorption or hydrogen bonding-dependent interactions are not considered in such cases. Researchers have previously discussed this and directly shown that non-covalent adsorption should also be taken into account, even though that the carbon material is functionalized using the covalent crosslinker-based approach [43–45]. Indeed, it is well-known that the hydrogen-bonding network and/or salt formation have significant influence on, for example, the shift of the absorption band coming from carboxyl moieties (IR analyses). Although scholars [40] have presented further studies on the functionalized GO and RGO materials, the structures of the modified graphene-family nanoplatforms constitute major inaccuracies.

### A comment on the functionalization of sp^2^ carbon: diazotization

The diazotization reaction is a widely applied protocol for the functionalization of sp^2^ carbon in the graphene sheet [9,46–48]. This phenomenon is desirable due to the commercial accessibility of the reactants (aromatic amines). The diazotization reaction is also a versatile approach, as a wide range of arylamines (bearing various substituents) can be subjected to the process [9].

**Figure 7:**
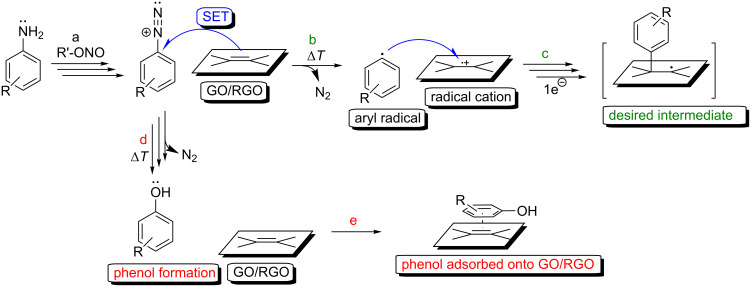
Mechanism of the covalent functionalization of graphene-family material applying diazonium salts chemistry: (a) generation of the diazonium salt, (b) single electron transfer (SET) between diazonium salt and graphene-family material, (c) radical addition, (d) formation of phenol, (e) adsorption of phenol onto the graphene sheet.

This reaction’s mechanism is not fully understood, but several researchers have discussed the reaction pathway [49–50]. Most plausibly, the reaction can be mainly attributed to rapid reactions based on electron-transfer processes. The first step of the diazotization reaction involves the generation of a diazonium salt from the corresponding amino reagent using a nitrite species (Figure 7, step a). Then (most likely) the aryl radical is obtained from the diazonium salt via the single electron transfer (SET) process and the inclusion of a graphene sheet (Figure 7, step b). This reaction step results in nitrogen extrusion. The desired functionalization route is most plausibly followed by a reaction between the generated radical species (Figure 7, step c), which is based on the addition of aryl radicals to the graphene sheet. One common approach is to conduct such a functionalization in an organic solvent and to use amyl or isoamyl nitrite to generate the diazonium salt. *o*-Dichlorobenzene is the most commonly used solvent in radical processes because it is not reactive toward radical species. Several researchers have reported protocols for functionalizing graphene-family materials using an organic solvent as the reaction medium and amyl nitrites as the additive [51–52]. There are also examples of a radical treatment of a graphene-family material in an aqueous environment with water-soluble sodium nitrite as the additive [53–57]. It is well-known that, in an aqueous environment, a diazonium salt undergoes a side reaction that results in the formation of phenolic compounds from the aryl radicals (Figure 7, step d); this formation can be considered to take place before the desired SET process with the graphene sheet (Figure 7, step b).

Importantly, it has been well documented that carbon materials are some of the best known adsorbents of phenolic compounds [58–61]. Water should therefore be considered a solvent that facilitates the adsorption of phenols on a graphene-family material during a reaction. This side process, which is visualized in Figure 7, step e, can also influence the reaction rate of the desired radical process. The diazotization approach utilizing amyl nitrites in organic solvent (e.g., *o*-dichlorobenzene) can therefore (i) enable an efficient electron-transfer process (Figure 7, step b), (ii) facilitate the desired reaction pathway (Figure 7, step c), and (iii) increase the functionalization yield. This approach is highly recommended in applications for sp^2^ functionalization that use the diazonium salts’ chemistry.

## Conclusion

With the growing number of articles on the application of graphene-family materials, a proper and rational design of a functionalization route is of the highest importance. Many scholars have employed the basic laws of organic chemistry to the covalent functionalization of graphene-family materials. These research works showcase that the chemistry of graphene, which includes many areas of science, both fulfils and heavily relies on the principles of organic synthesis. However, some of the works that are focused on graphene’s organic chemistry still contain major misunderstanding and inaccuracies. This synopsis includes a major discussion on the crucial role that water molecules and coupling reagents play in covalent modification processes. Special consideration was also given to the applied reactants, and in-depth analysis of obtained material’s structure is also of the highest importance. This work includes recommendations for the proper application of the basic organic synthesis principles for the functionalization of graphene-family materials.
